# Interspecific Phylogenic Relationships within Genus *Melilotus* Based on Nuclear and Chloroplast DNA

**DOI:** 10.1371/journal.pone.0132596

**Published:** 2015-07-13

**Authors:** Hongyan Di, Zhen Duan, Kai Luo, Daiyu Zhang, Fan Wu, Jiyu Zhang, Wenxian Liu, Yanrong Wang

**Affiliations:** 1 State Key Laboratory of Grassland Agro-ecosystems, College of Pastoral Agriculture Science and Technology, Lanzhou University, Lanzhou City, China; 2 College of Pastoral Agriculture Science and Technology, Lanzhou University, Lanzhou City, China; Saint Mary's University, CANADA

## Abstract

*Melilotus* comprises 19 species, while the phylogenetic relationships between species remain unclear. In the present work, three chloroplast genes, *rbc*L, *mat*K, *trn*L-F, and one nuclear region, ITS (internal transcribed spacer) belonging to 48 populations of 18 species of *Melilotus* were sequenced and phylogenetic trees were constructed to study their interspecific relationships. Based on the phylogenetic tree generated in this study using *rbc*L analysis, the *Melilotus *genus is clearly monophyletic in the legume family. Both Bayesian and maximum-parsimony approaches were used to analyze the data. The nrDNA ITS provided more informative characteristics (9.8%) than cpDNA (3.0%). *Melilotus* contains two closely related groups, clade I and clade II. *M*. *spicatus*, *M*. *indicus *and *M*. *segetalis* have a close relationship. *M*. *infestus*, *M*. *siculus* and *M*. *sulcatus* are closely related. The comparing between molecular phylogeny and flower color classification in *Melilotus *showed that the flower color is not much informative for phylogenetics of this genus.

## Introduction

*Melilotus* (sweet clover) belongs to the tribe Trifolieae of the legume family and comprises 19 annual and biennial species [1]. All species are native to Eurasia or North Africa [2], and three species are cultivated: *M*. *albus*, *M*. *officinalis* and *M*. *indicus* [3]. *M*. *albus* and *M*. *officinalis* are mainly capable of self-pollination [4], but when the pistil is longer than the stamens, there is very little self-pollination [5]. *Melilotus* also has entomophilous flowers, which can lead to hybridization. Several species have invaded the Northwest Territories in Canada and the Midwestern USA, among which *M*. *albus* and *M*. *officials* are often studied [6, 7, 8]. Members of the *Melilotus* genus have high seed yields and, relative to most other forages, are more tolerant to extremes in environmental conditions, e.g. drought, cold and high salinity [9, 10]. *Melilotus* also has important medicinal value in addition to being an important forage crop [11]. Furthermore, the nitrogen fixation rate of *Melilotus* is higher than that of other legumes, making it beneficial for crop rotations [12].

Members of *Melilotus* exhibit wide variations in flower structure, flower color, seed, leaf and pod characteristics [13, 14]. The classification of *Melilotus* are more difficult based on morphological traits and growth habits [15, 16]. However, except for morphological studies, no other taxonomic assessments have been conducted on interspecific phylogenetic relationships among species. Analysis of DNA has been widely used in the phylogenetic and classification studies. These methods are more effective and specific than traditionally morphological methods in phylogenetic relationships and genetic variation involved in sibling species and morphologically intermediate species [17, 18].

Phylogenetic results that used a single gene may lead to misleading, especially in cpDNA, which is inherited maternally [19]. Hybridization between different species or genera may lead to reticulate evolution [20]. The employment of a different molecular marker could help to assess and to reduce this problem. Nuclear ribosomal genes with alternating gene and spacer regions and tandom repeat structures can provide this option [21, 22, 23]. The nrDNA internal transcribed spacer (ITS) region and chloroplast DNA have higher variability and are thus suitable for classifying lower taxonomic levels [24, 25, 26]. Accordingly, these regions are useful for inferring phylogenetic relationships at lower taxonomic levels and have been successfully used to analyze plant systematics [27, 28]. Here we selected three cpDNA termed the *rbc*L gene, *mat*K gene and *trn*L-F gene and one nrDNA ITS to study the interspecific relationships [29, 30].

In this study, except for *M*. *macrocarpus* in *Melilotus* genus, plant samples from 48 populations of 18 *Melilotus* species were collected. To study the phylogenetic relationships among members of the *Melilotus* genus and to generate more accurate estimates of its genetic diversity, we constructed the molecular phylogenetic trees of single nrDNA ITS, 3-cpDNA and the concatenated sequences of all four genes. Finally the molecular phylogenetic classification was compared based on flower color and karyotype in *Melilotus*.

## Materials and Methods

### Sampling

Seeds from 48 populations representing 18 species were obtained from National Plant Germplasm System (NPGS, America) and planted at Yuzhong (35°57'N, 104°09'E) in Gansu Province, China (Table 1). Samples were collected from public land instead of protected areas in the northwest China, and no samples of endangered or protected species were included in our study.

**Table 1:** Information for 48 populations of 18 *Melilotus* species.

Species	Population number	Origin	Latitude	Longitude
***M*. *albus***	PI 90557	China, Manchuria	45°19'	124°29'
	Ames 21597	Italy	41°52'	12°34'
***M*. *altissimus***	Ames 18376	United States, Nebraska	41°29'	-99°54'
	PI 275975	-	-	-
	PI 420163	France	46°13'	-2°12'
***M*. *dentatus***	PI 108656	Armenia	40°4'	-45°2'
	PI 90753	China	35°51'	-104°11'
***M*. *elegans***	PI 250873	Iran	32°25'	-53°41'
	PI 260271	Ethiopia, Shewa	9°9'	-37°48'
***M*. *hirsutus***	Ames 22882	Russian Federation	61°31'	105°19'
	PI 129697	Sweden	60°7'	-18°38'
***M*. *indicus***	Ames 24055	Egypt	26°49'	30°48'
	PI 107562	Uzbekistan	41°22'	64°35'
	PI 260756	Turkey	38°57'	35°14'
	PI 43595	-	-	-
	Ames 21619	United States, Nebraska	41°29'	-99°54'
	PI 308524	Peru	-9°11'	-75°0'
***M*. *infestus***	PI 306327	Italy	41°52'	12°34'
	PI 306328	Hungary	47°9'	19°30'
	PI 306326	Algeria	27°13'	2°29'
***M*. *italicus***	PI 317638	Israel	31°2'	34°51'
	PI 317635	Czechoslovakia	14°28'	121°2'
***M*. *officinalis***	PI 304530	Turkey	38°57'	-35°14'
***M*. *polonicus***	PI 314386	Former Soviet Union	24°47'	120°60'
	PI 108647	Former Soviet Union	24°47'	120°60'
***M*. *segetalis***	PI 317633	Algeria	27°13'	2°29'
	PI 43597	-	-	-
	PI 317649	Czechoslovakia	14°28'	121°2'
***M*. *siculus***	PI 129703	Malta	35°56'	14°22'
	PI 318508	Greece	39°4'	21°49'
	PI 33366	Former Soviet Union	24°46'	120°59'
***M*. *speciosus***	PI 317650	Canada, Manitoba	53°45'	-98°48'
***M*. *spicatus***	PI 317644	Algeria	27°13'	2°29'
	Ames 25647	Ukraine, Krym	44°57'	34°6'
	Ames 18402	United States, Nebraska	41°29'	-99°54'
	PI 314466	Uzbekistan	41°22'	64°35'
***M*. *suaveolens***	Ames 18444	United States, Nebraska	41°29'	-99°54'
	Ames 23793	Mongolia	46°51'	103°50'
	PI 593408	United States, South Dakota	43°58'	-99°54'
	PI 595395	United States, lowa	41°52'	-93°5'
***M*. *sulcatus***	PI 198090	Morocco	31°47'	-7°5'
	PI 227595	Tunisia	33°53'	-9°32'
***M*. *tauricus***	PI 67510	Ukraine, Krym	44°57'	34°6'
	Ames 18446	United States, Nebraska	41°29'	-99°54'
	Ames 25789	Ukraine, Krym	44°57'	34°6'
***M*. *wolgicus***	PI 317665	Denmark	56°15'	9°30'
	PI 502547	Russian Federation	61°31'	105°19'
	PI 317666	Czechoslovakia	14°28'	121°2'

Young leaves from 2 to 12 individuals of each population were sampled (totaling 406 individuals). Leaves were frozen in liquid nitrogen and stored at -80°C.

### DNA extraction, amplification and sequencing

Four genes were amplified and sequenced: three chloroplast genes (cpDNA), *trn*L-F, *rbc*L and *mat*K, and one nuclear region (nrDNA), ITS (Table 2). For each population, 2 to 12 independent DNA samples were obtained to check for sequencing errors. Total genomic DNA was extracted using an SDS (sodium dodecyl sulfate) method [31]. Polymerase chain reactions were then conducted in a 25-μL tube containing 1 μL genomic DNA (50 ng / mL), 1 μL of each primer (5 pmol / mL), 12.5 μL Takara Taq DNA polymerase master mix and 9.5 μL deionized water. For nuclear DNA ITS, the region was amplified using a PCR protocol of 94°C for 3 min, followed by 35 cycles of denaturation at 94°C for 30 s, annealing at 50°C for 30 s, and extension at 72°C for 1 min, and a final extension at 72°C for 10 min. For *trn*L-F gene using a PCR protocol of 94°C for 3 min, then 30 cycles at 94°C for 45 s, annealing at 50°C for 45 s, extension at 72°C for 1min and a final extension step at 72°C for 7 min. The PCR temperature protocol of the *mat*K gene was: 94°C for 3 min then 35 cycles of denaturation at 94°C for 45 s, annealing at 58°C for 45 s, extension at 72°C for 1 min and a final extension step at 72°C for 10 min. Finally, for *rbc*L gene the following PCR conditions were used: initial denaturation at 94°C for 3 min, followed by 36 cycles of denaturation at 94°C for 30 s, annealing at 56°C for 30 s, and extension at 72°C for 1 min, and a final extension at 72°C for 10 min. The sequencing reactions were performed by Shanghai Shenggong Biotechnological, Ltd. (Shanghai, China).

**Table 2:** Sequences of primers used to amplify genes.

Primer	Sequence	Reference
**ITS_F**	GGAAGKARAAGTCGTAACAAGG	-
**ITS_R**	RGTTTCTTTTCCTCCGCTTA	-
***rbc*L_F**	AGACCTWTTTGAAGAAGGTTCWGT	[48]
***rbc*L_R**	TCGGTYAGAGCRGGCATRTGCCA	[48]
***mat*K_F**	CCCRTYCATCTGGAA ATCTTGGTTC	[49]
***mat*K_R**	GCTRTRATAATGAGAAAGATTCTGC	[49]
***trn*L-F_F**	CGAAATCGGTAGACGCTACG	[50]
***trn*L-F_R**	ATTTGAACTGGTGACACGAG	[50]

To analyze the phylogenetic relationship between *Melilotus* and other Legume forage and confirm the monophyly of genus *Melilotus*, we downloaded the only one available gene *rbc*L for most of legumes close to *Melilotus* from NCBI, including *Medicago*, *Trifolium*, *Caragana*, *Lathyrus* and *Vicia*. These *rbc*L sequences were used to construct a phylogenetic tree together with the sequences of 18 species of *Melilotus* obtained in the present study.

### Phylogenetic analyses

Phylogenetic analyses were performed using Bayesian and maximum-parsimony approaches. Sequence alignment was initially performed using ClustalX [32] and manually adjusted using MEGA5.0 [33]. The maximum-parsimony analyses involved a heuristic search strategy with 1000 replicates of random sequence addition in combination with TBR branch swapping in MEGA5.0. All character states were treated as unordered and equally weighted. Informative insertions and deletions (indels) were coded as binary characters (0, 1) according to Graham et al. (2000). A strict consensus tree was constructed from the most parsimonious trees. Bayesian analyses were conducted using MrBayes version 3.1 [34]. A model of sequence evolution for the combined dataset was selected using the program ModelTest version 3.6 [35] as implemented in MrMTgui [36] and based on the Akaike information criterion (AIC) [37]. The dataset was analyzed as a single partition using the GTR + I + G model. Four chains were run, beginning with a random tree and saving a tree every 100 generations for one million generations. Finally, the ITS region, three cpDNAs and the dataset of the four genes ITS, *rbc*L, *mat*K and *trn*L-F were combined for phylogenetic analyses. One sequence from each population was used to construct phylogenetic trees for the genus *Melilotus*.

## Results

### Alignments and DNA sequence data

A total of 96 haplotypes were identified for the ITS region, and 31, 83 and 106 haplotypes were identified for the *rbc*L, *mat*K, and *trn*L-F genes, respectively. One sequence from each population was used for constructing phylogenetic trees. Accession numbers of *rbc*L, *mat*K, *trn*L-F and ITS respectively are KP987625—KP987627, KP987673—KP987720, KP987577—KP987624 and KP987721—KP987768. The 755-bp fragment of the *rbc*L gene yielded the most parsimonious trees (length = 138 steps; CI = 0.667; RI = 0.928). The nrDNA tree was based on an alignment of 714 bp (length = 159 steps; CI = 0.765; RI = 0.929). The combined dataset of 3 cpDNAs comprised 2284 bp (length = 241 steps; CI = 0.838; RI = 0.916) and the 4-gene dataset 2998 bp, with maximum-parsimony analyses resulting in the most parsimonious trees (length = 252 steps; CI = 0.625; RI = 0.852). The aligned sequence information for the phylogenetic analysis is presented in Table 3.

**Table 3:** Dataset and tree statistics from separate maximum parsimony analyses.

Sequence	Number of sequences	Aligned Length/bp	Conserved site/bp	Variable site /bp	Singleton site/bp	Parsimony informative /bp	Parsimony informative site/%	(G+C) Content /%
***rbc*L**	42	755	666	88	27	61	8.1	40.8
**nrDNA**	48	714	572	111	42	69	9.8	49.0
**3—cpDNA**	48	2284	2058	117	29	88	3.0	35.0
**4-genes**	48	2998	2611	236	73	163	5.4	38.4

### Phylogenetic analyses

#### rbcL analysis in the legume family

**Fig 1:**
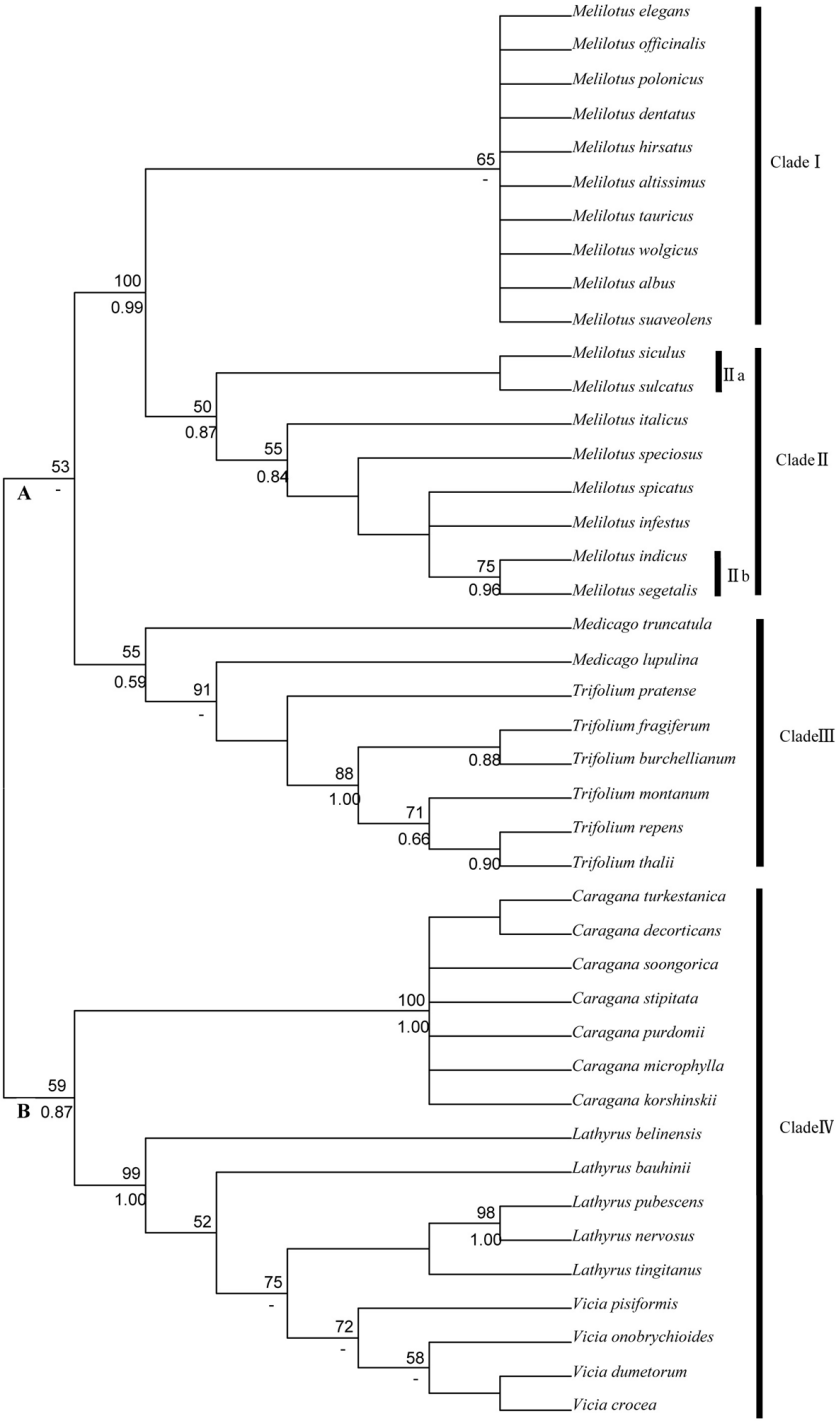
Topology resulting from maximum parsimony analysis of *rbc*L in the legume family dataset using MEGA5.0. Bootstrap support values (> 50%) are indicated above the branches (posterior values from the corresponding Bayesian analysis are provided below the branches;-: node not recognized).

The phylogenetic tree in the legume family shown in Fig 1 comprised two large clades, designated A and B. In phylogenetic tree, all the *Melilotus* species formed a monophyletic clade with a high bootstrap value of 100. Among them, the *Melilotus* was divided into two subclades, named clade I and clade II, with bootstrap values of 65 and 50, respectively. In clade II, *M*. *siculus* and *M*. *sulcatus* formed one subgroup, named clade IIa, and *M*. *indicus* and *M*. *Segetalis* formed another subgroup, named clade IIb. These *Melilotus* species clustering within the same subclade may have closer genetic relationships. All species of genera *Medicago* and *Trifolium* formed a clade named clade III which cluster together with genera *Melilotus* and can be used as outgroups in phylogenetic studies of *Melilotus*. All species of genera *Caragana*, *Lathyrus* and *Vicia* formed a big clade named clade IV, with members of *Lathyrus* and *Vicia* forming a subclade.

#### 3-cpDNA analysis

**Fig 2:**
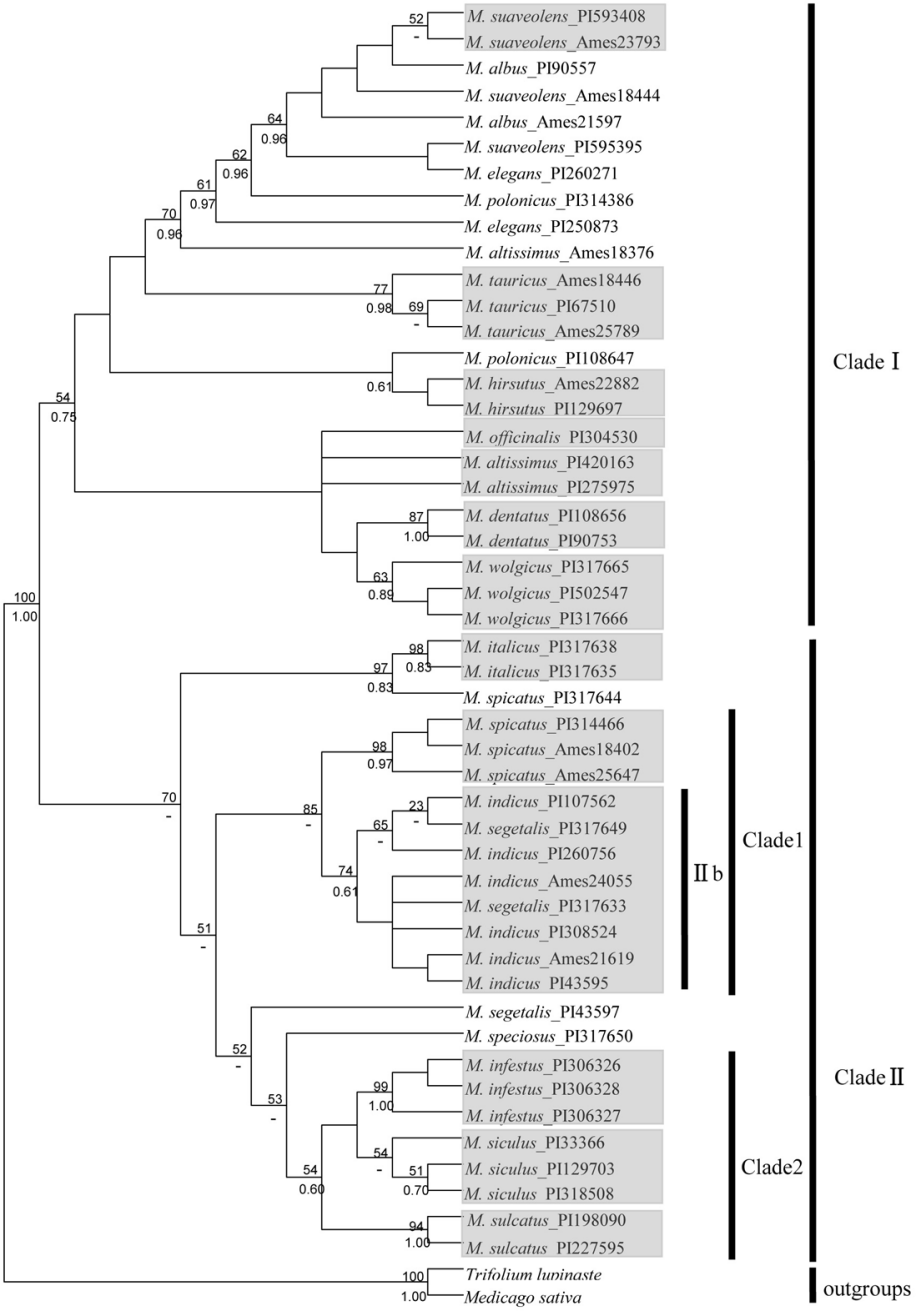
Topology resulting from maximum parsimony analysis of the combined dataset of 3-cpDNA genes (*rbc*L, *mat*K, *trn*L-F) using MEGA5.0. Bootstrap support values (> 50%) are indicated above the branches (posterior values from the corresponding Bayesian analysis are provided below the branches;-: node not recognized). The populations of the same species clustering together are indicated in a grey box. Except for clade 1 and clade 2, the definitions of clades follow those of Fig 1.

The 3-cpDNA tree of *Melilotus* based on 2284-bp of concatenated plastid sequences (*rbc*L, *mat*K and *trn*L-F), with *T*. *lupinaste* and *M*. *sativa* as outgroups, is shown in Fig 2. Similar to the *rbc*L tree for the legume family (Fig 1), the 3-cpDNA tree showed that *Melilotus* species can also be divided into two clades, clade I and clade II, though with low bootstrap support. In clade II, *M*. *spicatus*, *M*. *indicus* and *M*. *segetalis* formed a subclade named clade 1, which was supported by a high bootstrap value of 85; *M*. *infestus*, *M*. *siculus* and *M*. *sulcatus* formed clade 2, with low bootstrap values of 54. Compared with the results of the *rbc*L tree for the legume family, subgroup IIb was found in clade 1.

#### nrDNA analysis

**Fig 3:**
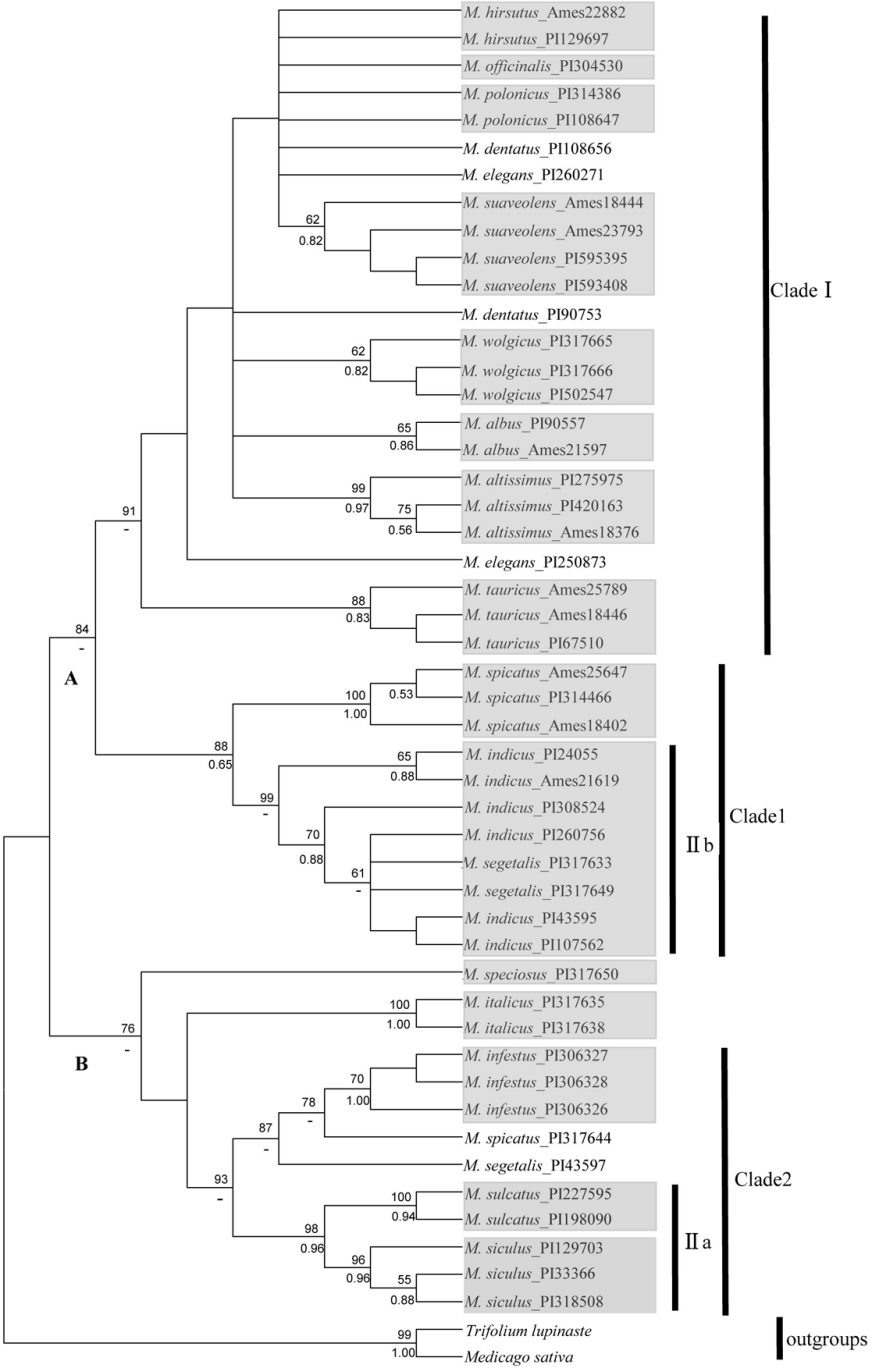
Topology resulting from maximum parsimony analysis of one ITS dataset using MEGA 5.0. Bootstrap support values (> 50%) are indicated above the branches (posterior values from the corresponding Bayesian analysis are provided below the branches;-: node not recognized). The populations of the same species clustering together are indicated in a grey box. The definitions of clades follow those of Fig 1 and Fig 2.

The ITS-based phylogenetic tree of *Melilotus* based on the 714-bp alignment is shown in Fig 3, with *T*. *lupinaste* and *M*. *sativa* as outgroups. Two strongly divergent and highly supported clades with bootstrap values of 84 and 76, respectively, are shown in the ITS tree. In contrast to the results in Fig 2, clade A consists of two subclades, clade I and clade 1, with high bootstrap values of 91 and 88. Within clade 1, *M*. *indicus* and *M*. *segetalis* form a well-supported subgroup named IIb. As shown in Fig 3, subgroup IIa is evidently within clade 2, and subgroup IIa includes two species, *M*. *sulcatus* and *M*. *siculus*, with high bootstrap support.

#### 4-gene analysis

**Fig 4:**
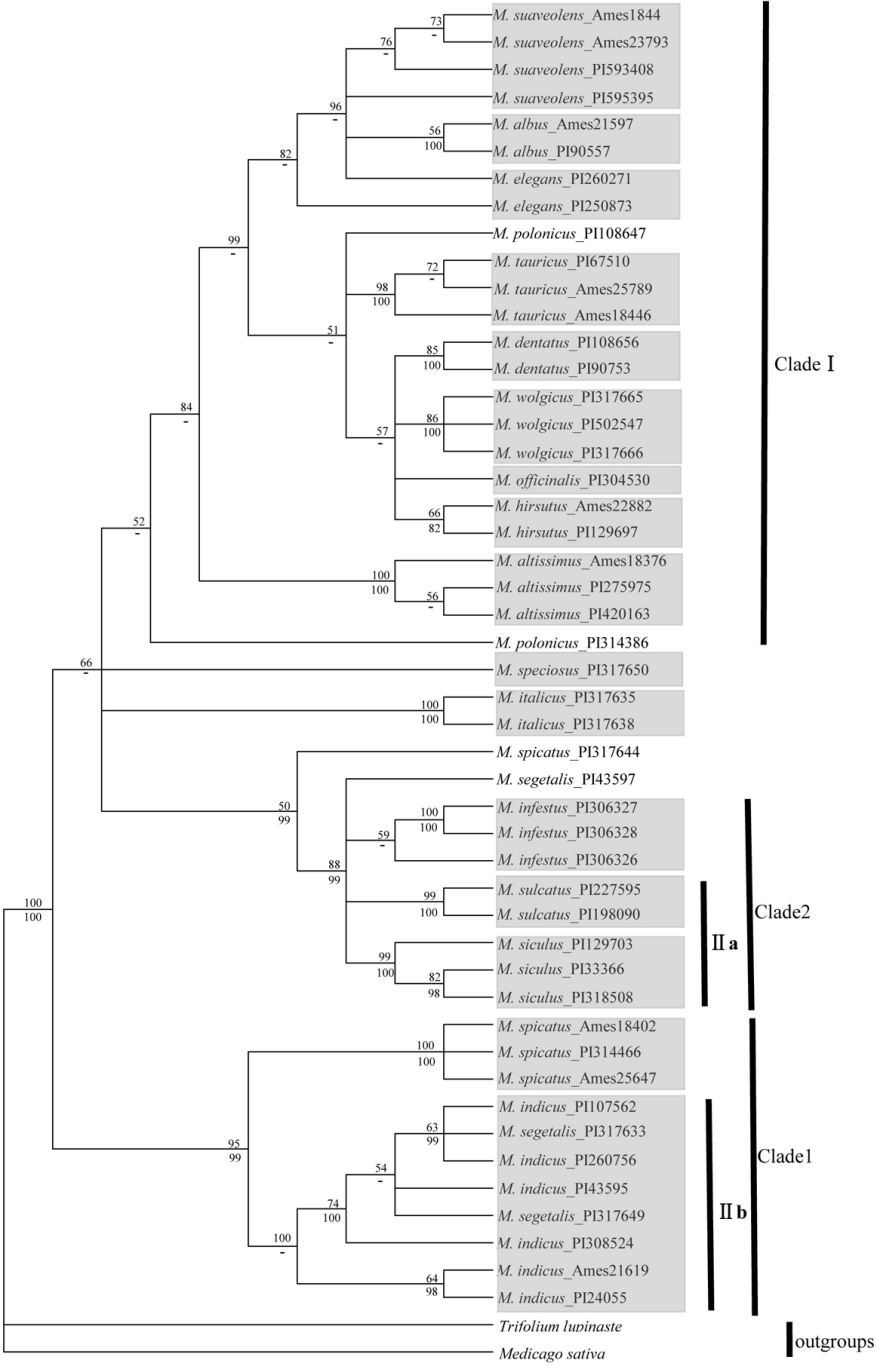
Topology resulting from maximum parsimony analysis of one 4-gene dataset using MEGA5.0. Bootstrap support values (> 50%) are indicated above the branches (posterior values from the corresponding Bayesian analysis are provided below the branches;-: node not recognized). The populations of the same species clustering together are indicated in a grey box. The definitions of clades follow those of Fig 1 and Fig 2.

The 4-gene tree of *Melilotus* yielded 2998 bp of four concatenated genes (*rbc*L, *mat*K, *trn*L-F and ITS), with *T*. *lupinaste* and *M*. *sativa* as outgroups, is shown in Fig 4. The major clades recovered in the above tree were also successfully resolved by this analysis. Clade I was observed and contained 10 related species. Subgroup IIa and *M*. *infestus* formed a subclade, namely clade 2, and subgroup IIb with *M*. *spicatus* formed a highly supported subclade, clade 1. However, clade 2 clustered together with clade I, which is the major difference between this tree and the nrDNA tree. Except for populations of *M*. *polonicus*, *M*. *spicatus* and *M*. *segetalis*, other populations of the same species formed a subclade in the 4-gene tree.

## Discussion

In this study, clade I, which contains 10 species, was found in all four trees of ITS and cpDNA genes. However, in the two cpDNA trees (Fig 1 and Fig 2), the 8 species formed another large clade, clade II. In the nrDNA tree (Fig 3) and 4-gene tree (Fig 4), clade 1, which was clustered into clade II in other two trees, was clustered into clade I; therefore, no clade II was shown. Several species are closely related in 3-cpDNA tree, nrDNA tree and 4-gene tree, e.g., *M*. *spicatus*, *M*. *segetalis* and *M*. *indicus* in clade 1, and *M*. *siculus*, *M*. *sulcatus* and *M*. *infestus* in clade 2. As shown in Table 3, the ITS region provided more informative characteristics (9.8%) than cpDNA (3.0%). Liu *et al*. [38] reported a similar result for Ligularia–Cremanthodium–Parasenecio in a study showing that ITS (39.6%) had more parsimony-informative characters than cpDNA (2.5%) using an *NdhF* and *trn*L-*trn*F combination. The higher sequence variability in the ITS region compared with cpDNA, which has also been demonstrated in many other taxa [39, 40, 41, 42] may lead to incongruence in phylogenetic tree. As nrDNA is biparentally inherited and has high rates of intraspecific gene flow which can enhance species delimitation. Howevre, the maternally inherited chloroplast DNA is more frequently introgressed and more limited use in species delimitation than nuclear DNA [43, 44]. In addition, incomplete lineage sorting [45] and hybridization between and within species [20] may also cause phylogeny incongruent.

According to Steven [16], plant morphology may show great variation within a single plant, which was not used for species classification. Steven studied agronomic and taxonomic reviews of the genus *Melilotus* and divided *Melilotus* into two groups according to flower color, namely, white and yellow. The white group contains four species, *M*. *albus*, *M*. *tauricus*, *M*. *wolgicus* and *M*. *speciosus*, and the other species compose the yellow group (Table 4). Our results showed that flower color has no obvious link with the phylogenetic classification in our study.

**Table 4:** The four classifications of flower color, seed morphology, karyotype and molecular phylogeny in *Melilotus*.

Categories	Classification	Subclassification		Species
**Flower color[11]**	white			*M*. *albus*, *M*. *tauricus*, *M*. *wolgicus*, *M*. *speciosus*
	yellow			*M*. *altissimus*, *M*. *dentatus*, *M*. *hirsatus*, *M*. *officinalis*, *M*. *polonicus*, *M*. *suaveolens*, *M*. *elegans*, *M*. *spicatus* ^*※*^, *M*. *indicus*, *M*. *segetalis*, *M*. *infestus*, *M*. *siculus* ^*※*^, *M*. *sulcatus*, *M*. *italicus*
**Karyotype[39, 40]**	Type A			*M*. *albus*, *M*. *altissimus*, *M*. *dentatus*, *M*. *hirsutus*, *M*. *officinalis*, *M*. *polonicus*, *M*. *suaveolens*, *M*. *tauricus*, *M*. *wolgicus*
	Type B	B-1		*M*. *elegans*, *M*. *indicus*, *M*. *neapolitana* ^*※*^
		B-2		*M*. *infestus*, *M*. *macrocarpus*, *M*. *messanensis* ^*※*^, *M*. *segetalis*, *M*. *speciosus*, *M*. *sulcatus*
	Type C			*M*. *italicus*
**Molecular phylogeny**	Clade **Ⅰ**			*M*. *albus*, *M*. *altissimus*, *M*. *dentatus*, *M*. *hirsatus*, *M*. *officinalis*, *M*. *polonicus*, *M*. *suaveolens*, *M*. *tauricus*, *M*. *wolgicus*, *M*. *elegans*
	Clade **Ⅱ**	Clade 1		*M*. *spicatus* ^*※*^
			Ⅱb	*M*. *indicus*, *M*. *segetalis*
		Clade 2		*M*. *infestus*
			Ⅱa	*M*. *siculus* ^*※*^, *M*. *sulcatus*
				*M*. *speciosus*, *M*. *italicus*

^*※*^: Species incongruence in flower color, karyotype and molecular phylogeny

Clarke studied the number and morphology of chromosomes in the genus *Melilotus* [46], reporting a chromosome number of 2n = 16. Karyotype analyses of all *Melilotus* species were conducted by Kita [47]. The 19 species examined are grouped into three types: A, B and C. Type B is further divided into Type B-1 and Type B-2 (Table 4). The grouping information based on karyotype analyses indicates that the species within each type are closely related [47]. Except for *M*. *elegans*, clade 1 of the phylogenetic trees is consistent with type A, and clade 2 comprises all Type B and Type C species. The molecular phylogeny classification in our study well support the karyotype classification. The better consistency between molecular phylogenetic and karyotype indicate that karyotype may be the significant phylogenetic signal in the *Melilotus* genus.

However, the phylogeography of *Melilotus* species and populations which rely on their distributions around the world remains largely unknown. Genetic diversity analysis within *Melilotus* genus is on going in our group with SSR markers, which will also provide a supplement conclusion of the interspecific relationship.
