# Correction: Interspecific Phylogenic Relationships within Genus *Melilotus* Based on Nuclear and Chloroplast DNA

**DOI:** 10.1371/journal.pone.0225421

**Published:** 2019-11-13

**Authors:** Hongyan Di, Zhen Duan, Kai Luo, Daiyu Zhang, Fan Wu, Jiyu Zhang, Wenxian Liu, Yanrong Wang

The Data Availability statement for the published article [1] states that all relevant data are within the paper. Here the authors clarify that the sequencing data are deposited at GenBank. There is an error in the accession numbers reported in the Results section of the article. The correct accession numbers of *rbc*L, *mat*K, *trn*L-F and ITS, respectively, are KP987625-KP987672, KP987673-KP987720, KP987577-KP987624 and KP987721-KP987768.

There is a previously published article by some of the same authors [2] that was not cited and discussed in the article. The previous study looked at genetic diversity in two species, *Melilotus albus* and *Melilotus officinalis* based on ITS and *trn*L-*trn*F sequences. There is no overlap in the *Melilotus* accessions used in the two studies.
